# Neurological effects of inorganic arsenic exposure: altered cysteine/glutamate transport, NMDA expression and spatial memory impairment

**DOI:** 10.3389/fncel.2015.00021

**Published:** 2015-02-09

**Authors:** Lucio A. Ramos-Chávez, Christian R. R. Rendón-López, Angélica Zepeda, Daniela Silva-Adaya, Luz M. Del Razo, María E. Gonsebatt

**Affiliations:** ^1^Departamento de Medicina Genómica, Instituto de Investigaciones Biomédicas, Universidad Nacional Autónoma de MéxicoMexico, DF, Mexico; ^2^Laboratorio Experimental de Enfermedades Neurodegenerativas, Instituto Nacional de Neurología y NeurocirugíaMexico, DF, Mexico; ^3^Departamento de Toxicología, Centro de Investigación y Estudios AvanzadosMexico, DF, Mexico

**Keywords:** arsenic, gestational, neurological effects, xCT, EAAC1, GLT1, NMDAR

## Abstract

Inorganic arsenic (iAs) is an important natural pollutant. Millions of individuals worldwide drink water with high levels of iAs. Chronic exposure to iAs has been associated with lower IQ and learning disabilities as well as memory impairment. iAs is methylated in tissues such as the brain generating mono and dimethylated species. iAs methylation requires cellular glutathione (GSH), which is the main antioxidant in the central nervous system (CNS). In humans, As species cross the placenta and are found in cord blood. A CD1 mouse model was used to investigate effects of gestational iAs exposure which can lead to oxidative damage, disrupted cysteine/glutamate transport and its putative impact in learning and memory. On postnatal days (PNDs) 1, 15 and 90, the expression of membrane transporters related to GSH synthesis and glutamate transport and toxicity, such as xCT, EAAC1, GLAST and GLT1, as well as LAT1, were analyzed. Also, the expression of the glutamate receptor N-methyl-D-aspartate (NMDAR) subunits NR2A and B as well as the presence of As species in cortex and hippocampus were investigated. On PND 90, an object location task was performed to associate exposure with memory impairment. Gestational exposure to iAs affected the expression of cysteine/glutamate transporters in cortex and hippocampus and induced a negative modulation of NMDAR NR2B subunit in the hippocampus. Behavioral tasks showed significant spatial memory impairment in males while the effect was marginal in females.

## Introduction

Experimental, as well as epidemiological studies, provide evidence suggesting that both environment and genetics are important components in the development of neuropathologies at early age or later in life. Diet components and chronic exposure to heavy metals and metalloids have been associated with the manifestation of neurological impairments, particularly when exposure occurs during the development and maturation of the nervous system (Winneke, 2011).

Inorganic arsenic (iAs) is an ubiquitous metalloid that is used in wood preservation, as a pesticide, in electronic devices due to its semiconductor capacities and also as a chemotherapeutic agent (ATSDR, 2007). This metalloid which is considered an epidemiologically important natural pollutant can be found in arsenic-containing minerals, ores and groundwater. Globally, more than 200 million of individuals drink water with levels of iAs above the World Health Organization reference value of 10 μg/L. Increased concentrations of iAs have been found in groundwaters in Argentina, Chile, China, India, Mexico, Taiwan and the USA where people are chronically exposed to iAs by drinking water from contaminated wells as a result of geothermal activities, mineral dissolution or deposition and weathering of atmospheric volcanic particles.

Deficits in cognitive functions as evidenced by decreased intelligence, verbal coefficients (Calderón et al., 2001) and impairments in learning and memory (Tsai et al., 2003; Rosado et al., 2007; von Ehrenstein et al., 2007; Asadullah and Chaudhury, 2008; Wasserman et al., 2014) have been associated with chronic exposure to iAs. The neurological and cognitive dysfunctions seem to be dependent on the concentration, timing and duration of exposure (Tyler and Allan, 2014).

In human and in many mammalian species iAs is reduced, methylated into trivalent and pentavalent methylated species and conjugated with glutathione (GSH, L-γ-glutamyl-L-cysteinyl-glycine, Thomas et al., 2004; Kumagai and Sumi, 2007). These events are associated with the generation of oxidative stress (Kumagai and Sumi, 2007). The presence of iAs and its methylated metabolites have been reported in umbilical cord blood studies from populations at risk (Concha et al., 1998; Parajuli et al., 2013) suggesting that they can cross the placenta and reach the developing fetus. An increasing number of epidemiological and animal model studies have shown that iAs exposure has harmful effects on brain function (Vahter, 2008; Parajuli et al., 2013; Tyler and Allan, 2014). However, there is little evidence of the neurotoxic effects during gestation a crucial development stage that may impact on normal adult life.

Studies in murine models have demonstrated that iAs crosses the blood-brain barrier (BBB) and is methylated in different brain regions that express the arsenic 3 methyltransferase (AS3MT) enzyme (Rodríguez et al., 2005; Sánchez-Peña et al., 2010). Transplacental transfer of As species from pregnant mice to fetus has been documented (Devesa et al., 2006; Jin et al., 2010). Moreover, AS3MT mRNA has been detected in mouse fetuses and embryos suggesting that As could be methylated in fetal tissues (Devesa et al., 2006). iAs methylation requires the presence of S-Adenosyl methionine as the methyl donor and cellular reductants such as thiorredoxin and GSH (Thomas et al., 2004). Thus, the metabolism of iAs consumes GSH, which is the main antioxidant in the central nervous system (CNS; Dringen, 2000). Inadequate GSH availability may modulate iAs biotransformation and determine disease susceptibility.

GSH penetrates the BBB poorly, therefore, CNS GSH levels depend on *de novo* synthesis which is limited by the intracellular availability of the sulfhydryl amino acid L-cysteine (L-cys; Valdovinos-Flores and Gonsebatt, 2012). Under aerobic conditions, L-cys autooxidizes to its disulfide form cystine (L-cys_2_), which is the predominant form of the aminoacid in plasma (Valdovinos-Flores and Gonsebatt, 2012). Specific membrane transporters such as xCT (SLC7A11)/4F2hc (SLC3A2), also known as the ${\text{x}}_{\text{c}}^{\text{−}}$ L-cys_2_/glutamate (L-glu) antiporter system, participates in the influx of L-cys for GSH synthesis (Valdovinos-Flores and Gonsebatt, 2012). xCT is widely expressed in both mouse and human brain (Burdo et al., 2006). The x_c_- system is also an important source of extracellular glutamate and is related to oxidative protection (Shih et al., 2006). However, because xCT uptakes L-cys_2_ in exchange for the excitatory L-glu, increased activity of this transporter could be deleterious and lead to excitotoxicity (Lau and Tymianski, 2010). The removal of extracellular L-glu involves EAAT3/EAAC1 (SLC1A1), part of the x-AG system in neurons and GLAST and GLT1 in glia. EAAC1 is also an important transporter for L-cys uptake in neurons (Valdovinos-Flores and Gonsebatt, 2012). Another important amino acid transporter system with wider substrate selectivity than ${\text{x}}_{\text{c}}^{\text{−}}$ or x-AG is the L system. LAT1 (SLC7A5) and LAT2 (SLC7A8) are the catalytic subunits of these amino acid transporters and are linked by a disulfide bridge to the heavy chain 4F2hc. *In vitro* and *in vivo* studies have provided evidence of L-cys transport by both LAT1 and LAT2 (Killian and Chikhale, 2001; Meier et al., 2002).

Glutamate is the most abundant excitatory neurotransmitter in the CNS. Its effects are mediated by ionotropic and metabotropic receptors. The ionotropic receptors (named after the agonists that activate them): α-amino-3-hydroxy-5-methyl-4-isoxazolepropionic acid (AMPAR) and N-methyl-D-aspartate (NMDAR) are widely expressed in the CNS. A distinct property of NMDAR is that allows the entry of Ca^2+^, in addition to the passage of K^+^ and Na^+^. Thus, excitatory postsynaptic potentials can increase Ca^2+^ levels in the postsynaptic neuron which can potentially act as a second messenger initiating signaling cascades. The activation of NMDAR is also voltage-dependent due to the extracellular blockage by Mg^2+^ or Zn^2+^. Then, the passage of cations (mostly Ca^2+^) occurs when the blockage is removed by a large number of excitatory inputs or the repetitive firing of the presynaptic cell or both. These properties are considered the bases of synaptic plasticity, learning and memory storage processes.

NMDAR is formed by several protein subunits producing a number of receptor isoforms. The expression of NMDAR subunits is differentially regulated during development and in response to synaptic activity. NR1/NR2A containing NMDAR receptors predominate at synaptic sites in the adult nervous system whereas NR1/NR2B receptors predominate during development and tend to be concentrated at extrasynaptic sites (Paoletti et al., 2013). NR2B subunits modulate the pharmacological and functional properties of the NMDA receptor (Mony et al., 2009). Consequently, NR2B has been implicated in modulating the synaptic function in activities such as learning, memory processing, and feeding behaviors, as well as being involved in a number of human disorders (Mehta et al., 2013). Additionally, the results of some experimental models suggest that exposure to xenobiotics might interfere with the expression of NMDAR subunits NR2A and NR2B during brain development (Olney et al., 2000; Li et al., 2012).

Studies using C3H and CD1 mice show that iAs crosses the placenta modifying gene expression that could lead to aberrant gene expression later in life (Shen et al., 2007; Waalkes et al., 2007). We hypothesized that the gestational exposure to iAs would up-regulate GSH *de novo* synthesis and L-cys_2_ influx via xCT and EAAC1 in brain cells. This condition could lead to increased levels of extracellular L-glu and to the modulation of NMDAR expression in brain regions such as cortex and hippocampus, where this receptor participates in learning and memory. Adult CD-1 male and female mice received 20 mg/L of iAs in drinking water for 1 month before mating. Pregnant females received continued exposure during gestation and lactation. At weaning, 50% of the pups continued drinking water with iAs while the rest drank deionized water similar to control animals. Results suggest that arsenic exposure disrupts L-cys and L-glu transport in the hippocampus by the up-regulation of xCT and EAAC1 and down-regulation of GLT1. This altered L-cys and L-glu transport was associated to the negative regulation of NR2B subunits and to impaired spatial memory.

## Materials and methods

### Chemicals

All chemicals were purchased from Sigma-Aldrich (St Louis, MO, USA) unless otherwise indicated. For western blots, primary rabbit antibodies against xCT, EAAC1, GLAST or GLT1 (ab37185, ab124802, ab416 or ab41621 respectively) were obtained from Abcam, Cambridge, MA, USA. Anti-LAT1 (sc-34554) from Santa Cruz Biotechnology, Santa Cruz, CA, USA. Rabbit anti-NR2A, anti-NR2B or mouse anti-GAPDH (AB1555P or AB1557P, MAB374 respectively) from Millipore, Bedford, MA, USA. Rabbit anti mouse-β-tubulin (T4026) from Sigma-Aldrich. Secondary goat anti-rabbit antibodies were obtained from Cell Signaling Technology (Danvers, MA, USA). For immunofluorescence staining chicken anti-MAP2 (ab5392) from Abcam. Anti-rabbit Alexa Fluor 594 (A11039) and anti-chicken Alexa 488 (A21207) secondary antibodies were obtained from Life Technologies, Carlsbad, CA, USA.

### Animals and treatment

Seven- to eight- week-old CD-1 mice were obtained from the Animal Care Facility at the Instituto de Investigaciones Biomédicas, UNAM, and were maintained at 23–25°C under a 12 h light/dark cycle and a relative humidity of 50–60%. Animals had free access to food (Harlan 2018S Diet; Harlan, Indianapolis, IN, USA) and water. Mice were housed in groups of 4 animals per plastic cage and separated by sex. One group of randomly selected mice (12 male and 12 female) received 20 mg/L of (iAs) daily as sodium arsenite via their drinking water for 30 days (exposed group). The same number of animals were assigned to the control group and received drinking water without iAs. The dose of treatment was chosen taking into consideration reports on iAs reproductive toxicity (Golub et al., 1998). Sodium arsenite solutions were prepared freshly daily in deionized water. After 30 days of treatment, each male was mated with one (single) female. Initiation of gestation was estimated by vaginal plug formation. Then, the males were removed, and the female mice were housed individually. Throughout the experiment, water consumption was recorded daily. Body weight was recorded every 6 days during the 30 days prior to mating in both sexes, and on days 0, 7, 14 and 18 of gestation in females. The exposed females continued to receive water with 20 mg/L of iAs during the gestation and lactation period. On postnatal days (PNDs) 1 and 15 randomly selected iAs exposed and control pups from each litter were sacrificed. On PND 1 whole brains were removed. On PND 15 the brain regions could be identified and were dissected on ice to isolate cortex and hippocampus. Tissue samples were immediately frozen by immersion in liquid nitrogen and maintained at −70°C until processed. Sex differentiation in the offspring was performed based on anogenital length (Suckow et al., 2000). On PND 15 the exposed litter was divided: 50% continued to receive drinking water with iAs while the rest received deionized water similar to control animals until PND 90. These 3 groups, including controls (Control), the group exposed to iAs only during gestation and lactation (iAs-PND 15) and the group exposed to iAs during gestation, lactation and for the first 90 days (iAs-PND 90) were used for the behavioral tests. Control litters continued to drink water without iAs. At weaning, on PND 21, mice in each litter were separated from the mothers.

The experiments were performed following the guidelines stated in the “Principles of Laboratory Animal Care” (NIH publication #85-23, revised 1985) and “Especificaciones técnicas para la producción, cuidado y uso de los animales de laboratorio (Clave NOM-062-ZOO-1999)” of the “Norma Oficial Mexicana de la Secretaría de Agricultura, Ganadería, Desarrollo Rural, Pesca y Alimentación (SAGARPA)” (published August, 2001).

### Western blots

Membrane enriched fractions were obtained from frozen tissue samples as described previously (Schindler et al., 2006) for western blot determination of xCT, EAAC1, LAT1, GLAST, GLT1 and the NMDA receptor subunits NR2A and NR2B. Briefly, frozen tissues were homogenized in 10 volumes of extraction buffer containing 10 mM HEPES, 10 mM NaCl, 1 mM KH_2_PO_4_, 5 mM NaHCO_3_, 5 mM EDTA, 1 mM CaCl_2_, 0.5 mM MgCl_2_, 1 mM PMSF, and 10 mg/ml aprotinin and leupeptin. The homogenates were centrifuged at 6,300 g for 10 min at 4°C. Supernatants were recovered and centrifuged at 100,000 g for 30 min at 4°C. The pellets were finally suspended in 40 mM Tris-HCl, pH 9.5, 8 M urea and 4% (w/v) Triton X-100. Protein concentrations were determined using a Pierce BCA Protein Assay kit (Thermo Scientific, Meridian Rd, Rockford, USA). The samples (5–40 μg protein per well) were subjected to SDS-PAGE and transferred into nitrocellulose membranes (Bio-Rad Laboratories, Germany). The membranes were blocked with TBS containing 5% Blotto and 0.1% Tween-20 and incubated with the respective primary antibodies. The blots were probed with mouse anti-β-tubulin or anti-GAPDH after stripping, which were used as loading controls. The protein bands were visualized with appropriated HRP-linked secondary antibodies using the ECL Prime western blotting detection reagent (GE Healthcare Bio-Sciences, Pittsburgh, PA); images were captured and densitometric analysis was performed with Image J software version 1.46r software (U. S. National Institutes of Health, Bethesda, Maryland, USA).

### Immunofluorescence

On PND 15, mice were transcardially perfused with ice-cold 0.9% saline followed by ice-cold 4% paraformaldehyde in phosphate buffer (PB), pH 7.4. Brains were removed, postfixed at 4°C and successively immersed in 20% and 30% sucrose cryoprotection solutions. Sections (22 μm) were collected in 24-well culture plates filled with 0.9% phosphate buffered saline (PBS), pH 7.4. After 3 washes with PBS + 0.3% Triton X-100 (PBST), the sections were incubated overnight at 4°C in rabbit anti-xCT (1:100) or rabbit anti-EAAC1 (1:300) and chicken anti-MAP2 (1:800) primary antibodies with a 2% normal horse serum in PBST solution. After 3 washes in PB solution sections were incubated with anti-rabbit Alexa Fluor 594 and anti-chicken Alexa 488 secondary antibodies (1:300) diluted in PB solution for 2 h. Finally, sections were mounted with Vectashield mounting medium with DAPI (Vector Laboratories, Burlingame, CA, USA) and analyzed under the microscope. Photomicrographs were acquired with an Olympus BX51WI DSU confocal microscope (Olympus, Center Valley, PA, USA) coupled to a Hamamatsu EM-CCD C9100 camera (Hamamatsu, Hamamatsu, Japan).

### GSH and GSSG level determination

Tissue GSH and GSSG levels were measured in whole brain, the cortex and hippocampus using a microplate-adapted fluorometric *o*-phthalaldehyde (OPA) method (Senft et al., 2000). Fluorescence was determined with 365 nm excitation and 430 nm emission filters in a DTX 800/880 Multimode Detector (Beckman Coulter, Fullerton, CA, USA).

### Methylated arsenic species determination

Concentrations of arsenic species were determined in urine, whole brain, the cortex and hippocampus by hydride-generation atomic absorption spectrometry using cryotrapping (HG-CT-AAS) as described previously (Hernández-Zavala et al., 2008). The quantification was performed using independent calibration curves of the arsenic species. Arsenic acid disodium salt (99% pure), and dimethyl arsinic acid (DMAV; 98% pure) were obtained from Sigma-Aldrich. Methylarsonic acid (MMAV) disodium salt (99% pure) was obtained from Ventron (Danvers, MA, USA). Sodium borohydride was obtained from EM Science (Gibbstown, NJ, USA). Prior to analysis, tissue samples were digested with 2 X ultrapure grade phosphoric acid (J. T. Baker) as described by Hughes et al. (2003).

### Behavioral test “place recognition task”

The object location task was conducted using the methodology described by Mumby et al. (2002), which was adapted to mice. Behavioral testing was performed in a 30 cm H × 60 cm W × 60 cm L acrylic open field box with white walls. Two objects of identical shape, size and color (white, black or red) were used in each trial. The objects and box were cleaned with a mixture of 10% ethanol, 10% dextran in destilled water, prior to each trial to eliminate any odor cues. Throughout the experimental period, the distal environmental cues were kept constant, and dark conditions were maintained in the experimental room. For three consecutive days, mice were habituated individually to the context and experimenter, by performing one session per day for 10 min. On day 4, the test was conducted in two phases: the exploration phase for recognition of object location, and the test phase, for discrimination of the location change. In the exploration phase, the two objects were placed in identical orientations with respect to two opposite corners of the box (10 cm from the corners) and the animals were allowed to explore the objects for 5 min. The animals were then returned to their homecage for 15 min and in the meantime the cage was cleaned and one of the objects was moved to a new location (opposite corner, at half the initial distance from the corner but maintaining an identical orientation with respect to the corner of the box). The animal was returned to the arena and let explore the objects for 5 min. The sessions were recorded with a video camera and analyzed by a trained observer. The behavior measurements included the frequency and cumulated time in seconds in which an animal approached (touch with body or vibrissae) and contacted each object with the paws during exploration. The discrimination index (DI) was calculated based on the formula DI = time spent exploring the re-placed object/time spent exploring both objects. Animals that explored the unmoved objects for less than 10 s during the test phase were not included in the study. DI was also determined in the exploration phase for each object, to verify object or place preference.

### Statistical analysis

The data are expressed as the means ± SE. The number of animals tested is indicated in each case. Student’s *t*-test or one way analysis of variance (ANOVA) were used to assess statistical significance followed by Dunnett’s multiple comparison test or Tukey’s *post hoc* test. A *p* value <0.05 was considered statistically significant in all cases.

## Results

### Arsenic exposure

**Figure 1:**
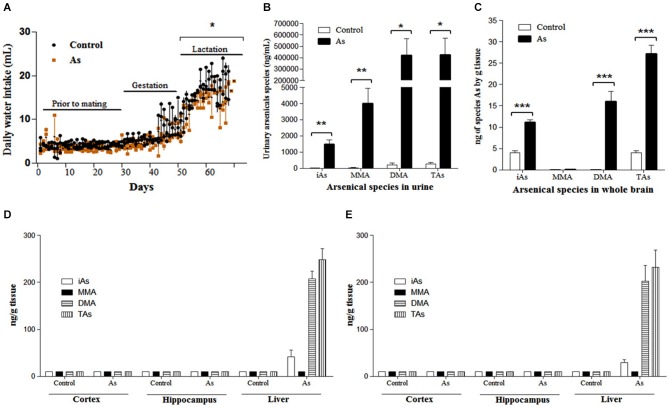
**Water intake and As species in urines and brain tissue (A) Water intake of pregnant females and their litters**. Daily water intake in was estimated by daily weighing the water bottles. Significant differences were observed only during lactation. **(B)** As species in pregnant female urine were determined in control and exposed females (*n* = 6) on days 15–18 of gestation. 24 h urine collection was performed using a metabolic cage. **(C)** iAs and As species in whole brain on PND 1 and PND 15 in male **(D)** and female **(E)** pups, when sex and brain regions could be identified. Accumulation of As was observed only in the liver during lactation, *n* = 6. One pup was randomly chosen from each litter. The values are expressed as mean ± SE; *P < 0.05, **P < 0.01, ***P < 0.001 vs. control group. iAs: inorganic arsenic, MMA: monomethylarsenic, DMA: dimethylarsenic, TAs (iAs + MMA + DMA): total arsenic.

Parental exposure to 20 mg/L of iAs during 30 days was well tolerated and did not alter body weight, water consumption or mating behavior (data not shown). No differences in body weight between control and exposed females were observed during gestation. A significant decrease in water consumption was observed in exposed female mice during lactation and until weaning (Figure 1A). The litter size between exposed and un-exposed groups was also similar (data not shown). The exposed litter did not show signs of overt toxicity, i.e., ataxia, redness, swelling, fetal malformations or death at birth and throughout the experiment. (Figure 1A). The estimated average intake of iAs for males was 2.69 ± 0.69 mg/kg per day during the 30 days prior to mating, while females ingested 2.72 ± 0.88 mg/kg/day. During gestation, the amount of iAs ingested was 2.92 ± 1.17 mg/kg/day (20–21 days), and during lactation the amount increased to 10.73 ± 1.9 mg/kg/day. A significant decrease in water intake was observed in the exposed group.

### Arsenic species in the urine of the mother and in the whole brain and regions of the offspring with gestational exposure on PND 1 and PND 15

As species levels were determined in the urine of pregnant females between days 15–18 of gestation. The main As species in exposed female urine was DMA followed by MMA and iAs. Total As levels among exposed females was 1500 times higher than that in controls (Figure 1B). On PND 1, the whole brain of the exposed litter showed DMA (58.9%) and iAs (41%) as the main As species. The levels of As species in control litters were 6.7 times lower than those of the exposed offspring (Figure 1C). During lactation (PND 15), the levels of As species in the male and female cortex and hippocampal regions were not different between controls and exposed mice (Figures 1D,E). As species accumulation was observed in the livers of exposed mice (Figures 1D,E).

### Effects of gestational exposure on GSH levels in whole brain on PND 1, and in the cortex and hippocampus on PND 15 mice

**Figure 2:**
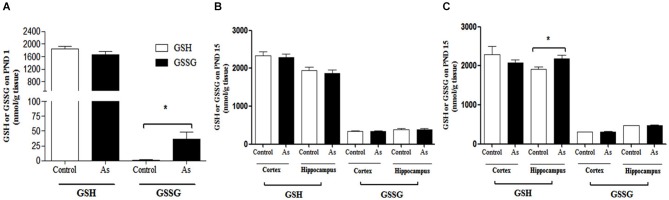
**GSH and GSSG levels in mouse brain on PNDs 1 and 15**. **(A)** On PND 1, As species were determined in the whole brain. **(B,C)** On PND 15 cortex and hippocampus were dissected from males and females and processed for GSH and GSSG determination. Data are expressed in mean nmol/g of fresh tissue ± SE. Data were analyzed using Student’s *t*-test. (*) Significantly different from controls, *P* < 0.05, *n* = 9 per group.

We hypothesized that the gestational exposure to iAs would modulate GSH levels in brain cells. On PND 1, the gestationally exposed litter showed significantly increased levels of oxidized GSH (GSSG) in the whole brain (Figure 2A). At this stage, sex or brain regions could not be clearly differentiated. On PND 15, no changes in GSH or GSSG levels were observed between the cortex and hippocampal regions from exposed and control male mice (Figure 2B). However, female mouse hippocampal regions showed a significant increase in GSH levels contents (Figure 2C).

### Changes in GSH levels induced by iAs exposure are associated with changes in the expression of xCT, EAAC1 and LAT1 transporters and modulation of NMDAR subunits in different brain regions

**Figure 3:**
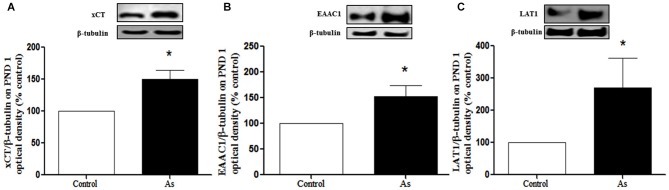
**Cystine/cysteine and glutamate transporter expression in controls and gestational exposed mice on PND 1**. Whole Brain was removed and processed for western blotting as described in Materials and Methods for **(A)** xCT, **(B)** EAAC1 and **(C)** LAT1 expression. Densitometric evaluation of the blot images was performed using β-tubulin as loading control. Bars represent mean ± SE relative to control values, *n* = 6 per group. Data were analyzed using Student’s *t*-test. (*) Significantly different from controls, *P* < 0.05. Representative blot images are shown.

**Figure 4:**
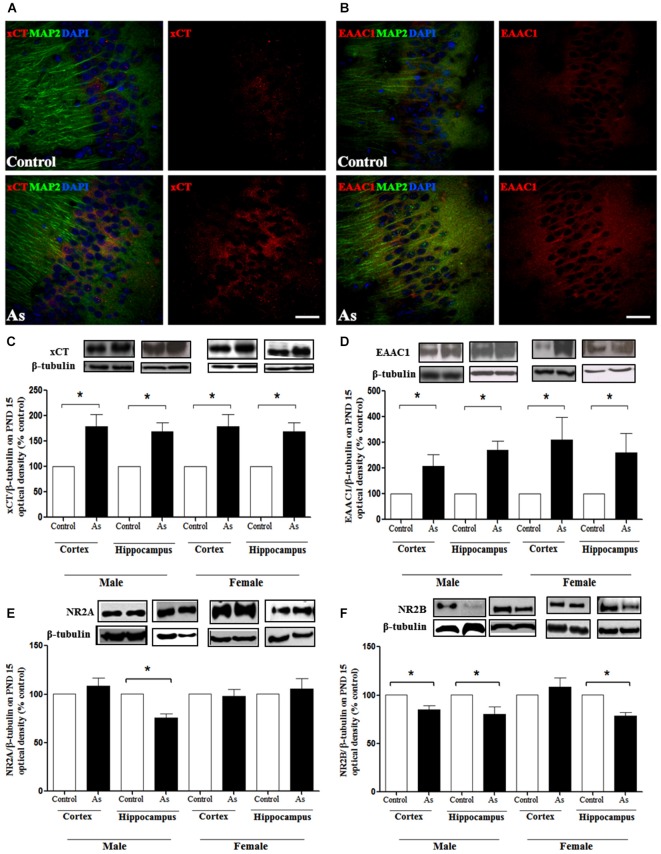
**Cystine/cysteine and glutamate transporters expression in control and gestational exposed mice on PND 15**. Confocal images of **(A)** xCT and **(B)** EAAC1 expression in control and PND 15 CA1 hippocampal cells. Neuron marker anti-MAP2 (green), xCT (red) or EAAC1 (red). Nucleus were counterstained with DAPI (blue). Scale bar, 30 μm. The cortex and hippocampus regions were removed from males and females and processed for western blotting as described in Materials and Methods for **(C)** xCT; **(D)** EAAC1;**(E)** NMDAR NR2A and; **(F)** NMDAR NR2B subunits. Densitometric evaluation of the blot images was performed using β-tubulin as loading control. Bars represent mean ± SE relative to control values, *n* = 4–6. Data were analyzed using Student’s *t*-test. (*) Significantly different from controls,*P < 0.05, **P < 0.01,***P < 0.001. Representative blot images are shown.

Changes in GSH could be due to the modulation of cysteine and glutamate transporters. Western blot analysis was performed to explore the expression of xCT, EAAC1 and LAT1 transporters in the whole brain on PND 1 and in the cortex and hippocampus on PND 15 and 90. The expression of xCT, EAAC1 and LAT1 transporters (Figures 3A–C respectively) was significantly increased in the brains of pups at PND 1. This up-regulation continued at PND 15 for xCT and EAAC1 (Figures 4C,D) but not for LAT1 (data not shown) in gestationally exposed male and female mice. The expression of xCT and EAAC1 was observed mainly in hippocampal neurons (Figures 4A,B). Increased extracellular levels of glutamate have been associated with modulation of NMDAR subunits. At the same time, iAs exposure down-regulated the NR2A NMDA receptor subunits in the male hippocampus in PND 15 pups (Figure 4E), while the NR2B subunit was down-regulated in both the male and female cortex and hippocampus (Figure 4F).

**Figure 5:**
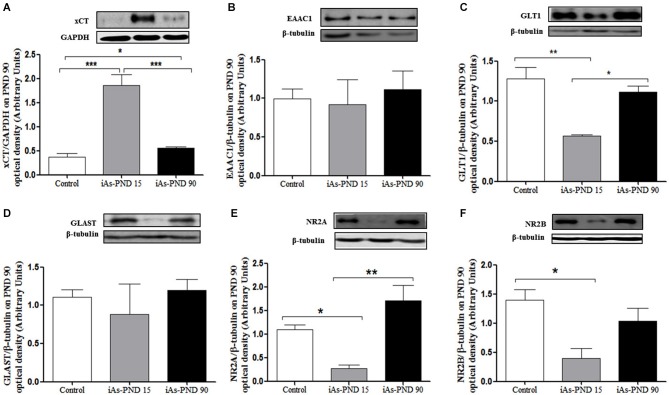
**Cystine, cysteine and glutamate transporters in male hippocampus on PND 90**. **(A)** xCT; **(B)** EAAC1;** (C)** GLT1; **(D)** GLAST; **(E)**NR2A; **(F)** NR2B. Densitometric evaluation of the blot images was performed using β-tubulin as loading control. Bars represent mean ± SE relative to control values. Data were analyzed using one-way ANOVA and Tukey’s *post hoc* test. (*) Significantly different from controls, *P* < 0.05., *n* = 3–5. Control: Controls, iAs-PND 15: exposure to arsenic during gestation and up to day 15th during lactation, iAs-PND 90: exposure to iAs during gestation, lactation and until day 90th.

On PND 15, gestationally exposed offspring were divided, and one half received drinking water with 20 mg/L of sodium arsenite while the other half received water without arsenic until PND 90. The place recognition task was performed at this time-point. The animals were then sacrificed and the hippocampal regions were examined for transporter expression. Exposed male showed increased xCT expression, although continued exposure (iAs-PND 90) seemed to significantly diminish the levels of xCT as compared with the animals that were only exposed during gestation and until PND 15 (iAs-PND 15; Figure 5A). No change in EAAC1 expression was observed in any of the groups (Figure 5B). However, the expression of GLT1 was down-regulated in the animals exposed during gestation and until PND 15 (iAs-PND 15), and no changes were observed in GLAST (Figures 5C,D). With respect to the NMDA receptor subunits, the expression of both NR2A and NR2B was significantly down-regulated in those animals exposed only during the gestation period and until PND 15 (iAs-PND 15; Figures 5C,D). Female mouse hippocampal regions showed marginal up-regulation of xCT expression in both conditions (iAs-PND 15 and -PND 90; *p* < 0.08) and non-statistically significant changes in the expression of GLAST, GLT1 or NMDAR subunits (data not shown).

### Arsenic gestational exposure disrupts the place recognition task performance “spatial memory”

**Figure 6:**
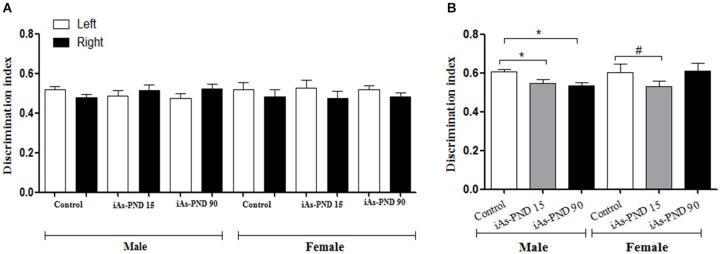
**The effect of As exposure on the spatial working memory in the recognition place task in male and female mice on PND 90**. Discrimination index (DI) = (novel place exploration time/total exploration time). **(A)** male and female DI of location in the recognition phase and **(B)** during test phase in controls, animals exposed during gestation and lactation (iAs-PND 15) and animals exposed during gestation, lactation and until PND 90 (iAs-PND 90). Each bar represents the mean ± SE (*n* = 8). Data were analyzed using an ANOVA. (*) Significantly different from controls after Dunnett’s *post hoc* test, *P* < 0.05. # *P* = 0.0625.

The place recognition task was used in 90 day old animals to determine if iAs exposure had an impact on the spatial memory, a function in which the hippocampal formation is strongly involved. The cognitive discrimination ability when an object changes location was evaluated in the different experimental groups of male and female mice including the control group, the iAs-PND 15 and iAs-PND 90 groups. During the recognition phase, no preference was observed for the location of the object or the object itself, which was measured as the time devoted to exploring each object (Figure 6A). In the test phase, iAs-PND 15 and iAs-PND 90 males showed significantly decreased recognition of the object location (Figure 6B). In contrast, this effect was marginally significant in iAs-PND 15 females and not significant in those with longer exposure (iAs-PND 90; Figure 6B).

## Discussion

iAs is an ubiquitous metalloid, present in man-made products as well as in food and water. The presence of iAs in drinking water causes health detrimental effects worldwide. Chronic exposure usually occurs through generations, however, few studies have investigated *in utero* developmental effects of iAs exposure (Vahter, 2008) while the impact at a molecular levels remains less understood. In exposed populations, As species can be present in cord blood (Concha et al., 1998; Hall et al., 2007) indicating that arsenic is transferred to the fetus. Moreover, the presence of As in cord blood has been inversely associated with neurodevelopmental indicators (Parajuli et al., 2013) and correlate with cognitive deficits that include alterations in pattern memory, functional memory, full scale IQ and verbal IQ (Tyler and Allan, 2014).

NMDA glutamate receptors participate in learning and memory. Overstimulation of these receptors by excess of glutamate can cause cell death during glucose and oxygen deprivation (Jung et al., 2012) and memory deficits due to xenobiotic exposure (Olney et al., 2000; Li et al., 2012). L-cys_2/_ L-glu, L-cys and L-glu transporters participate in GSH synthesis in the CNS and are modulated by oxidative stress. The hypothesis of this work was that exposure to iAs during gestation would increase oxidative stress up-regulating xCT. This condition might increase the efflux of L-glu that could lead to the activation of transporters to remove the extracellular glutamate. At the same time, oxidative stress and excess of glutamate might modulate NMDAR subunit expression (Scimemi et al., 2009) in the developing brain which might be reflected later as memory impairment.

Mice metabolize iAs and clear iAs metabolites from tissues more efficiently than humans (ATSDR, 2007). Results of the present study show that whole brain and liver tissues of mice exposed to 20 mg/L contained on average concentrations of total As species of 28 and 260 ngAs/g, respectively (Figures 1D,E). Similar concentrations have been found in liver samples from residents of an arsenic endemic area who drink water containing between 0.22–2 mg/L of iAs. In this case, human liver samples showed concentrations of 100–1,200 ngAs/g of liver tissue, respectively (Mazumder, 2005).

The levels of iAs in drinking water (20 mg/L) did not cause any visible signs of toxicity to mice before mating or during gestation in CD1 mouse. Control and exposed litters were similar in size (number of individuals), weight and sex composition. In contrast, in a similar study using the FVB/NJ mouse strain reduced fertility was observed (He et al., 2007) most likely due to different strain sensitivity. iAs exposed lactating females, significantly diminished water consumption (Figure 1A). To verify the iAs toxicokinetics, As species were determined in pregnant female urine and in the brain and liver tissues from the offspring. DMA was the main As species observed in exposed female urine during late gestation (Figure 1B) similar to results documented in human studies (Concha et al., 1998). In the newborn brains, iAs and DMA were the predominant species (Figure 1C). On PND 15, brain regions could be identified and the cortex and hippocampus were isolated. At this time, the levels of As species in exposed nursing males or females were not different from control males or females (Figures 1D,E). This was not the case for the liver where the main species was DMA. The As species accumulation after gestational exposure observed in this study was similar to that reported by Jin et al. (2010) for albino mice. It has been shown that As accumulation in milk is very low in humans (Concha et al., 1998). Thus, these results indicate that the presence of As in the newborn brains resulted from absorption through the placenta and was eliminated from the brain but not from the liver during lactation. This could reflect differences in the kinetics at the organ level that affect the disposition of As metabolites (Devesa et al., 2006).

The presence of iAs and DMA in the brain of newborn exposed litters suggests that the placenta does not limit the passage of As species and could be associated with the higher levels of oxidized GSH (GSSG; Figure 2A). At the same time, the transporter systems x_c_- (xCT), x-AG (EAAC1) and LAT1 were up-regulated (Figures 3A–C respectively) suggesting an increased in L-cys transport for GSH synthesis due to the elevation of GSSG. In lactating litters, (PND 15) the levels of GSH or GSSG in the cortex and hippocampus were not different from those determined in control animals (Figures 2B,C) most likely due to the lower levels of As species (Figures 1D,E). However, the up-regulation of xCT and EAAC1 transporters continued (Figures 4A,B) and was also observed in CA1 hippocampal cells by immunofluorescence (Figures 4A,B). Increased expression of both xCT and EAAC1 could protect against glutamate toxicity (Lewerenz et al., 2006) reducing NMDAR activation (Scimemi et al., 2009). At the same time, a significant down-regulation of the NR2B subunit in both sexes and NR2A subunit in the hippocampus of exposed PND 15 males but not females, was observed. This observation suggests that the alteration of L-cys and glutamate transport may modify NR2B expression in the hippocampus due to gestational exposure. Prenatal stress (Zhao et al., 2013), ethanol (Brady et al., 2013), high fat diets (Page et al., 2014) and nicotine (Wang et al., 2011) have been associated with down-regulation of NR2B or the NR2B/NR2A ratio. The reduced expression of NR2B in the NMDAR has been observed in connection with cognitive impairments and neuropathologies (Paoletti et al., 2013). On PND 90, exposed litters showed significantly impaired place recognition performance compared to controls. Males were more affected than females (Figure 6B), suggesting that hippocampal neurons were affected by As exposure. Animals were sacrificed to investigate transporters and NMDAR subunit expression on PND 90 hippocampus. xCT expression remained to be up-regulated (Figure 5A) especially in those animals exposed to iAs during gestation and until PND 15. However, on PND 90, EAAC1 expression was not different between control and exposed litters. GLT1, another transporter that participates in the removal of glutamate, was also down regulated in animals exposed during gestation. These results suggest that glutamate levels might be increased in the hippocampus of these mice, leading to NR2A and NR2B down-regulation (Figures 5C,D,F) and memory deficits (Figure 6B). These observations are consistent with findings where extracellular glutamate increase initiates adaptive responses that involve a gradual down-regulation of the expression of NMDA receptors in response to environmental toxics or a glutamate transport blocker in neuronal models (Cebers et al., 2001; Win-Shwe et al., 2009). PND 90 males exposed during gestation and that continued to drink water with arsenic also showed memory deficits, increased expression of xCT and marginal down-regulation of NR2B but not NR2A, suggesting that synaptic efficiency could be affected. Further research is needed to clarify these observations. Taken together, our data may support the idea of GSH as an important neuronal reservoir to prevent excitotoxicity (Koga et al., 2011).

Our results indicate that gestational exposure to iAs impairs NMDAR subunits expression in the hippocampus affecting spatial memory. This impairment is associated with increased oxidative damage at birth and altered L-cys_2_/ glutamate and L-cys transport, which might in turn down-regulate NR2B expression (Paoletti et al., 2013). NMDAR subunit expression changes during brain development. NR2B is more abundant during the second week of postnatal development as neurons mature and become enriched at extrasynaptic sites (Roullet et al., 2010; Qiu et al., 2011). Gestational and postnatal exposure disrupts this pattern down-regulating NR2B especially on PND15 when this subunit is more predominant. Mouse hippocampus EAAC1, does not alter the activation of receptors at the synaptic cleft but reduces the recruitment of NR2B-containing NMDAR in perisynaptic/extrasynaptic sites (Scimemi et al., 2009). Then, the increased expression of EAAC1 observed in iAs exposed mice, could further alter the glutamate lifetime in the extracellular space impairing NMDAR activation and the induction of long term potentiation.

Cognitive impairment was observed in the rat after realgar (a mineral drug containing arsenic) exposure. Excess of extracellular glutamate was observed in hippocampus. Glutamate accumulation in the synaptic cleft was related to decreased expression of NR1 and up-regulation of NR2A subunit leading to calcium overload, down-regulation of GLT1 and ultrastructural changes in hippocampal neurons (Tao-guang et al., 2014). Interestingly, increased activity of xCT transport is accompanied with an increase in glutamate levels and neuronal death via overstimulation NMDAR (Jackman et al., 2012). Also, xCT^−^/^−^ mice show significantly lower extracellular hippocampal glutamate concentrations and optimal spatial working memory (De Bundel et al., 2011) suggesting that xCT constitutes a source for non-vesicular glutamate release. Additionally, down-regulation of NR2B can occur through ubiquitination if NMDAR agonists are increased (Ehlers, 2003). Then, disrupted glutamate transport by increased xCT activity might be responsible for the down-regulation of NR2B. *In vitro* cultures of hippocampal neurons have shown that both NR2B and NR2A show endocytosis trafficking through endosomes (Scott et al., 2004). This event could be disrupted by prenatal As exposure. Also, adult rats exposed to different concentrations of sodium arsenite during 3 months after weaning, showed cognitive impairments and a dose-dependent down-regulation of NR2A in both mRNA and protein levels in hippocampus (Luo et al., 2009, 2012). Similarly, NR2A up-regulation was observed on PND 90 in male mouse hippocampus (Figure 5E). The NMDAR NR2A subunit in adult rat is sensitive to arsenic induced neurotoxicity (Luo et al., 2009, 2012).

Additionally, the persistent lower expression of NR2B subunit in males might be due to epigenetic changes. iAs methylation consumes GSH and SAM which affects DNA methylation (Reichard and Puga, 2010; Tyler and Allan, 2014). According to Reichard and Puga (2010) the epigenetic modifications observed during mouse gestational exposure to iAs suggest target gene-specific methylation changes, some of which are hypomethylated while others suffer hypermethylation. Thus, hypermethylation of NR2B promoter and/or hypomethylation of NR2B repressors could lead to the down-regulation of NR2B. In this respect, rats exposed during gestation to 3 and 36 ppm of sodium arsenite in drinking water showed changes in the methylation status of genes involved in neuronal plasticity in cortex and hipocampus (Martínez et al., 2011). Histone modifications have been also observed in mice prenatally exposed to 100 μg/L of sodium arsenite which could be associated with altered learning in adults (Cronican et al., 2013). Also, there are evidences that the changes in the activity-dependent NR2B expression (Lee et al., 2008) or NR2B expression during chronic intermitent ethanol treatment (Qiang et al., 2010) are due to epigenetic modifications.

The altered activity/expression of the NR2B subunit due to post-transcriptional modifications (Qiu et al., 2011), ubiquitinization (Ehlers, 2003) or epigenetic modifications (Lee et al., 2008; Qiang et al., 2010; Tyler and Allan, 2014) have been implicated in the modulation of learning and memory processing, pain perception, feeding behavior as well as being involved in neurological disorders. Our results show that NMDAR subunit expression by prenatal exposure to iAs is affected, which may in turn alter memory early in life, which is in line with what has been reported in some human populations (Tyler and Allan, 2014). Learning and memory are complex processes involving several brain regions and neuronal networks. This work shows how As species might disrupt the expression of key components that could lead to behavioral alterations and the development of neuropathologies later in life. It remains important to identify the environmental agents that might impair neural development, maturation and physiology by interfering with the biochemistry of crucial neurotransmiters and aminoacids such as glutamate and cysteine.

## Conflict of interest statement

The Guest Associate Editor Victoria Campos declares that, despite being affiliated to the same institution as author Daniela Silva-Adaya, the review process was handled objectively and no conflict of interest exists. The authors declare that the research was conducted in the absence of any commercial or financial relationships that could be construed as a potential conflict of interest.
